# Supplementary material for: Quantitative Proteomic and Phosphoproteomic Profiling of Lung Tissues from Pulmonary Arterial Hypertension Rat Model
Source: Int J Mol Sci. 2023 Jun 1;24(11):9629. doi: 10.3390/ijms24119629 (PMC10253736; doi:10.3390/ijms24119629)
Supplement: Supplementary file 1 [file ijms-24-09629-s001.zip › Supplementary Figures.pdf]

a

	Week 1	Week 2	Week 3	Week 4	Total
Proteins identified	5560	5496	5907	5547	6901
Proteins quantified	5398	5290	5737	5378	6759

b

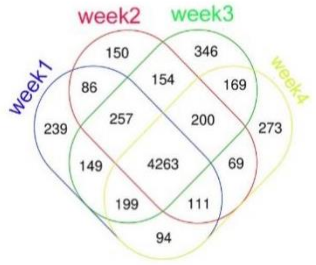

Supplementary Figure S1. Summary of protein numbers in proteomic profiling of lung tissues of MCT-treated rats and the control rats. a Table summarizing the numbers of proteins identified and quantified. **b** Venn diagram showing the protein numbers across four groups.

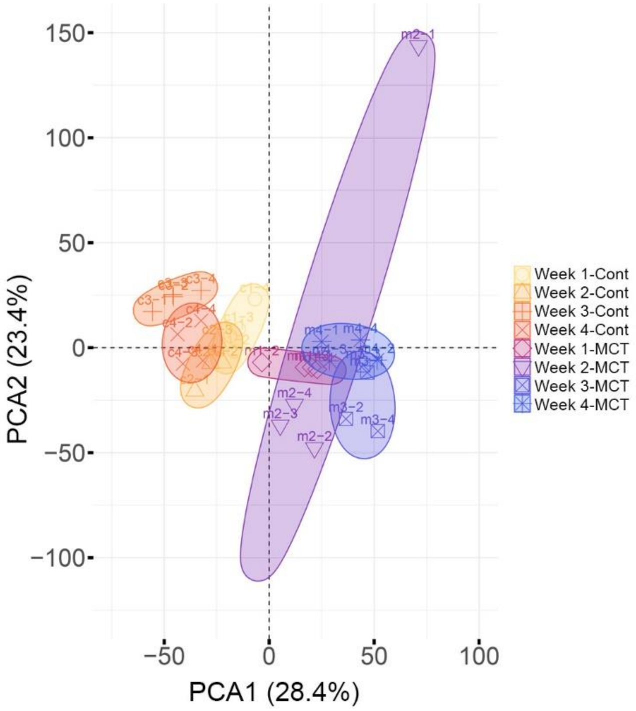

Supplementary Figure S2. Principal component analysis (PCA) of protein abundances for all biological replicates of control (Cont) and MCT treated samples

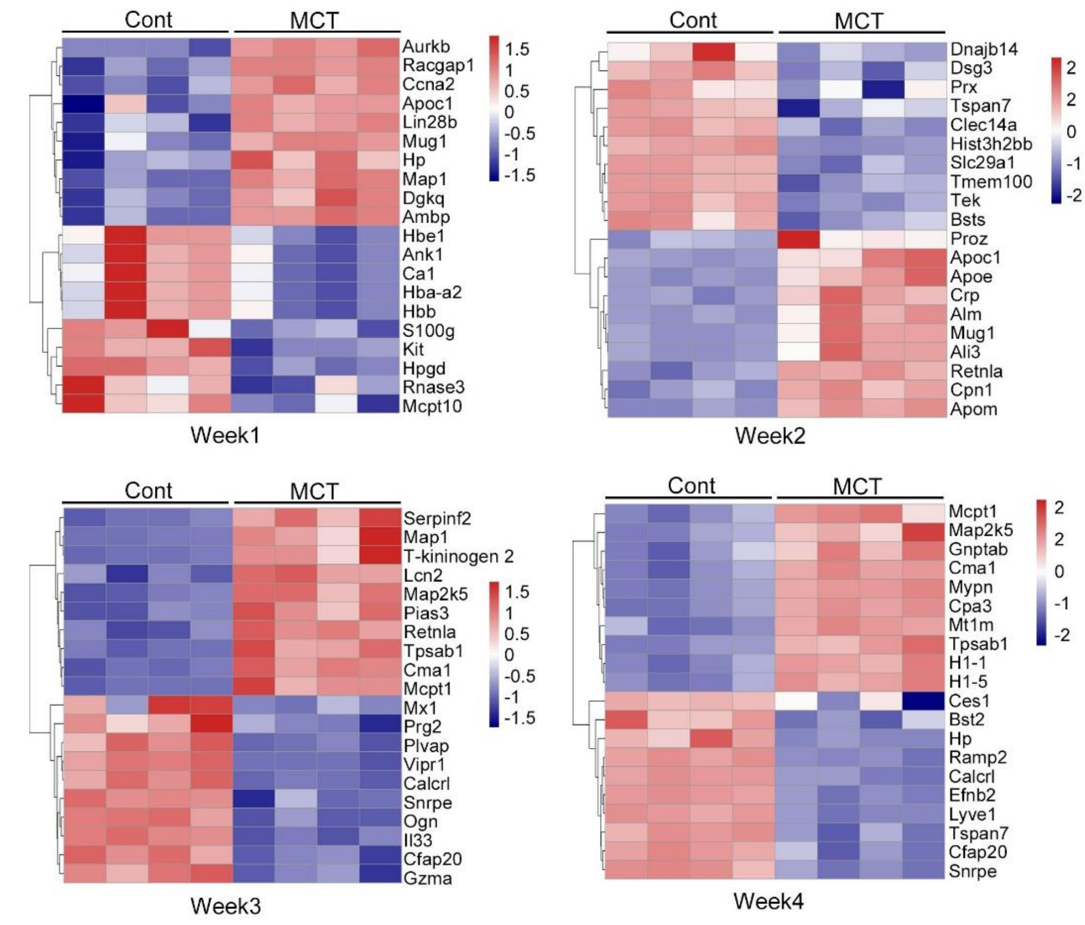

Supplementary Figure S3. Heatmaps showing the top-10 changed proteins in each group. Uncharacterized proteins were removed.

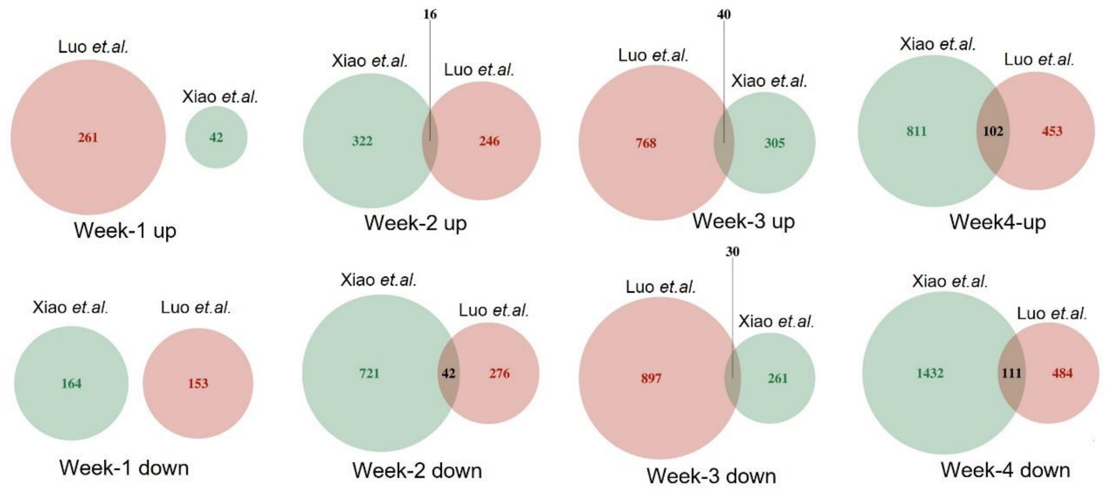

Supplementary Figure S4. Comparison of significantly changed genes or proteins identified by this study (*Luo et. al.*) and *Xiao et. al.*

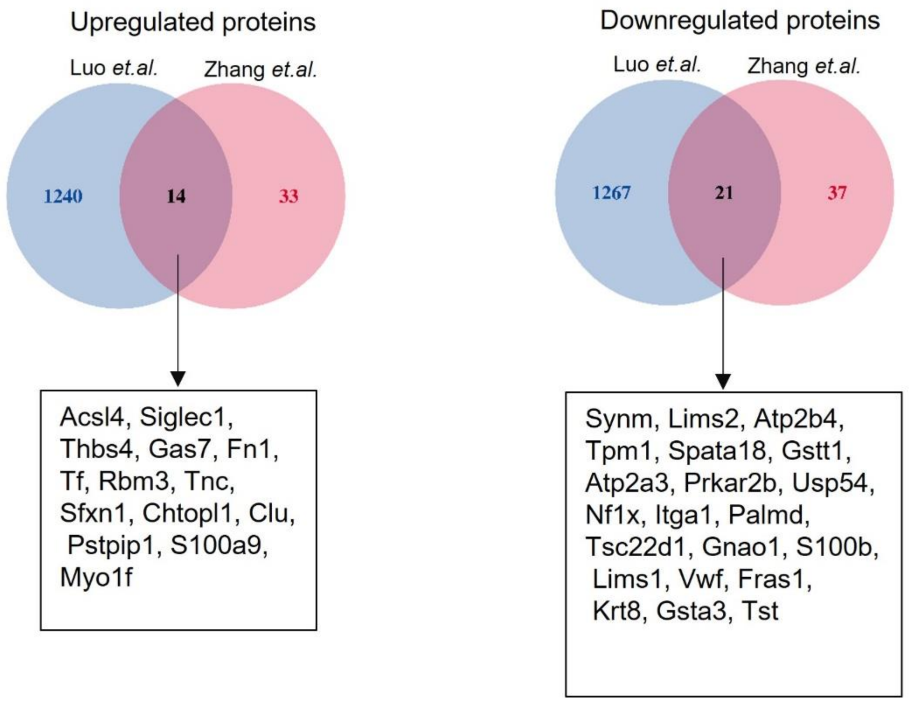

Supplementary Figure S5. Comparison of significantly changed proteins identified by this study (*Luo et. al.*) and Zhang *et. al.* All the significantly upregulated proteins or downregulated proteins identified in week 1,2,3 and 4 were pooled and compared with the data of Zhang *et. al*.

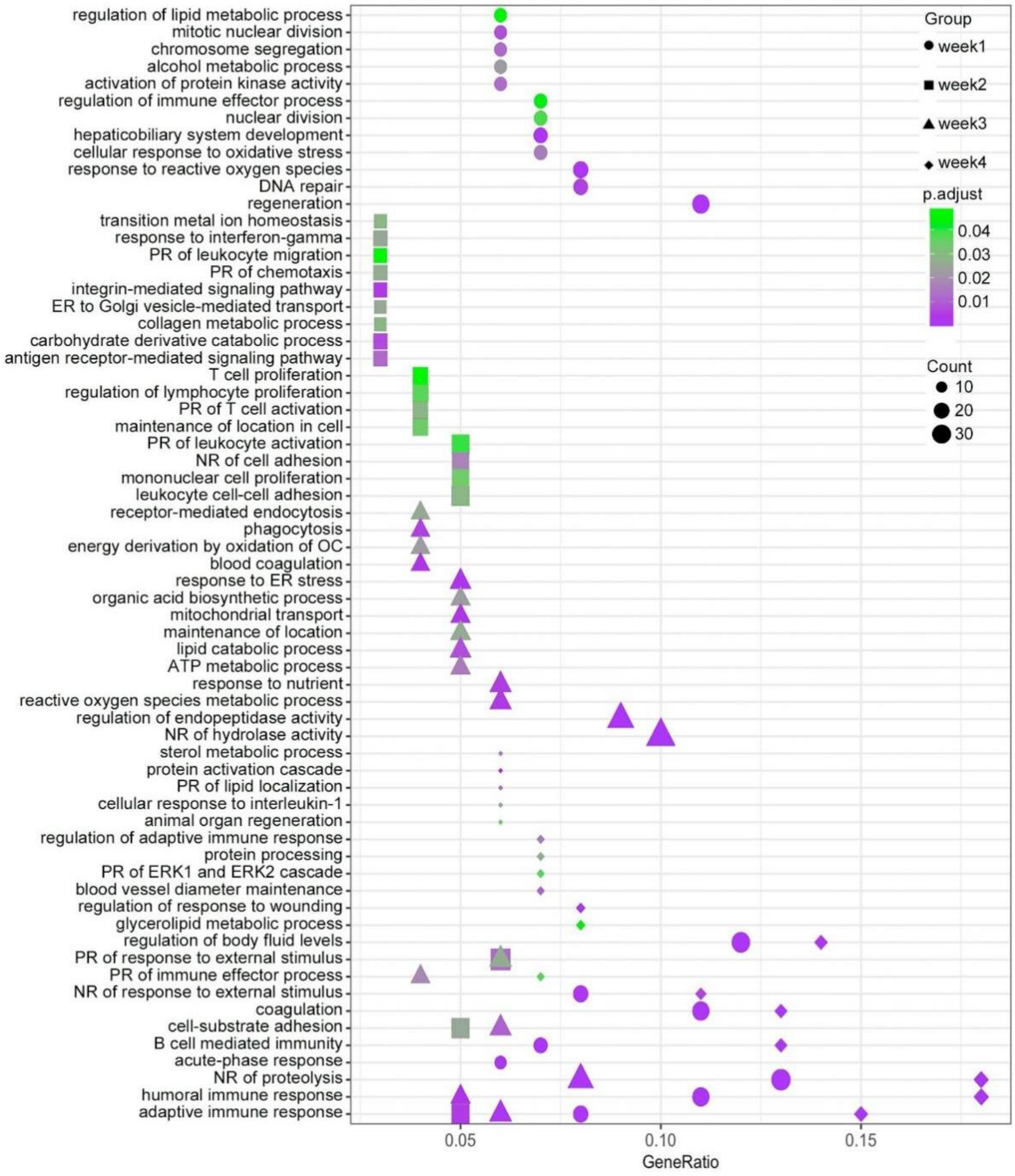

Supplementary Figure S6. GO (biological process) analysis of the proteins significantly upregulated in MCT samples comparing to control samples. For each group of data, top-20 terms were shown based on gene count. PR is short for positive regulation, and NR is short for negative regulation.

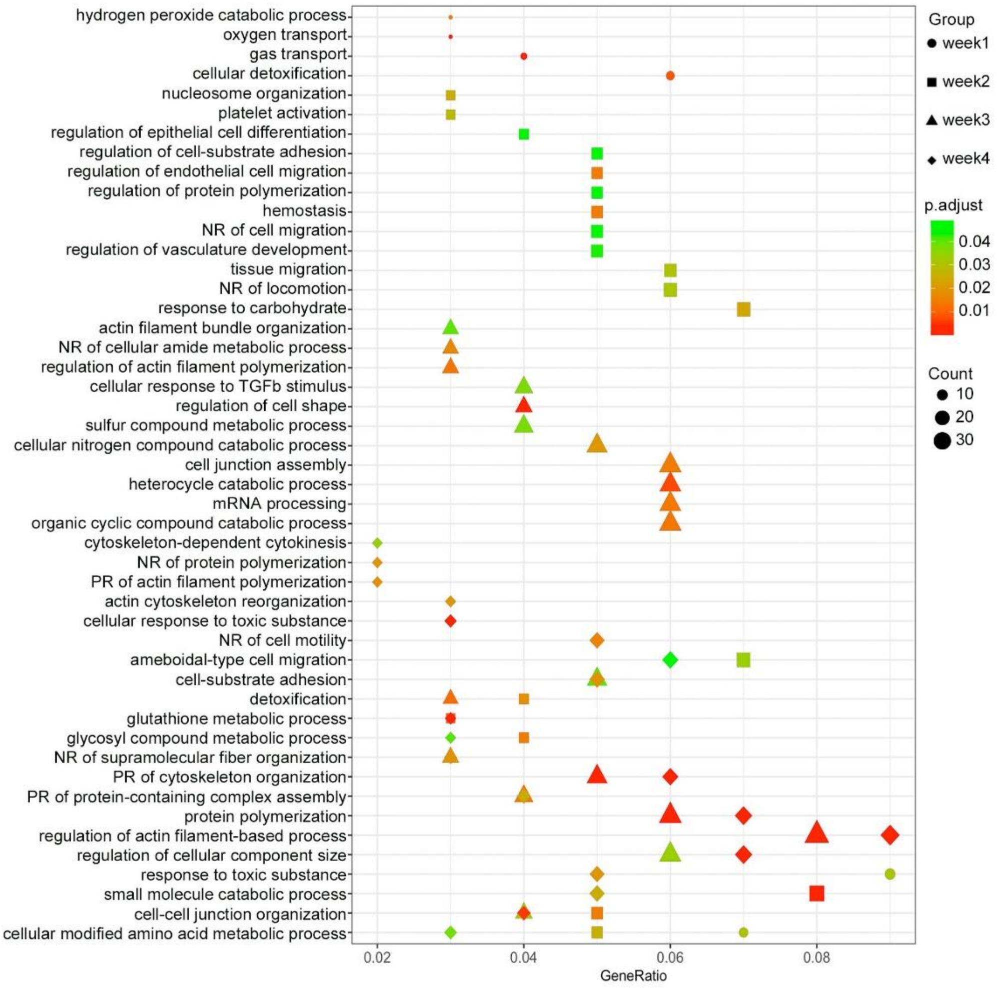

Supplementary Figure S7. GO (biological process) analysis of the proteins significantly downregulated in MCT samples comparing to control samples. For each group of data, top-15 terms were shown based on gene count. PR is short for positive regulation, and NR is short for negative regulation.

a

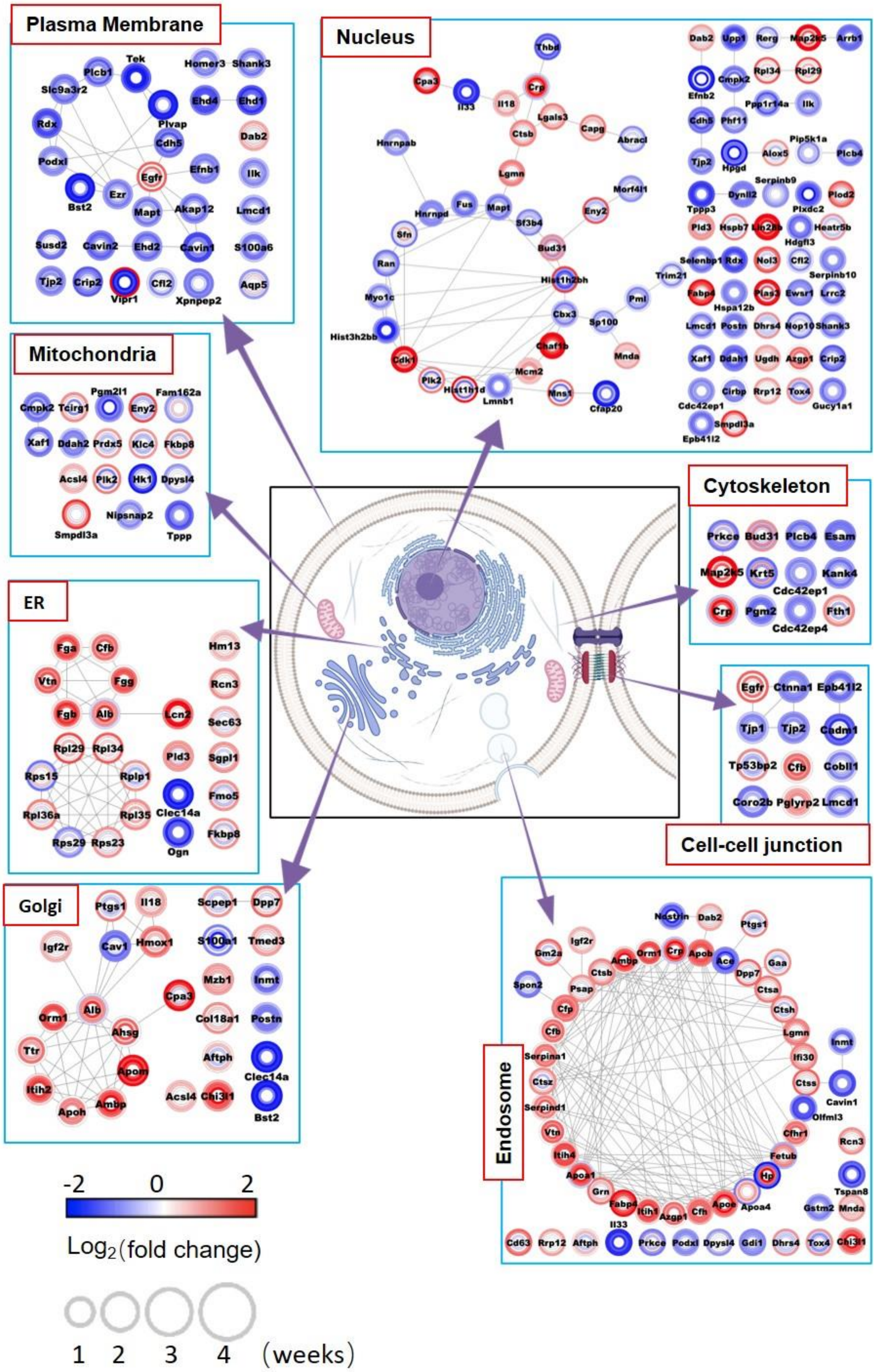
Plasma Membrane
Nucleus
Mitochondria
Cytoskeleton
ER
Cell-cell junction
Golgi
Endosome
-2
0
2
Log2(fold change)
1
2
3
4
(weeks)

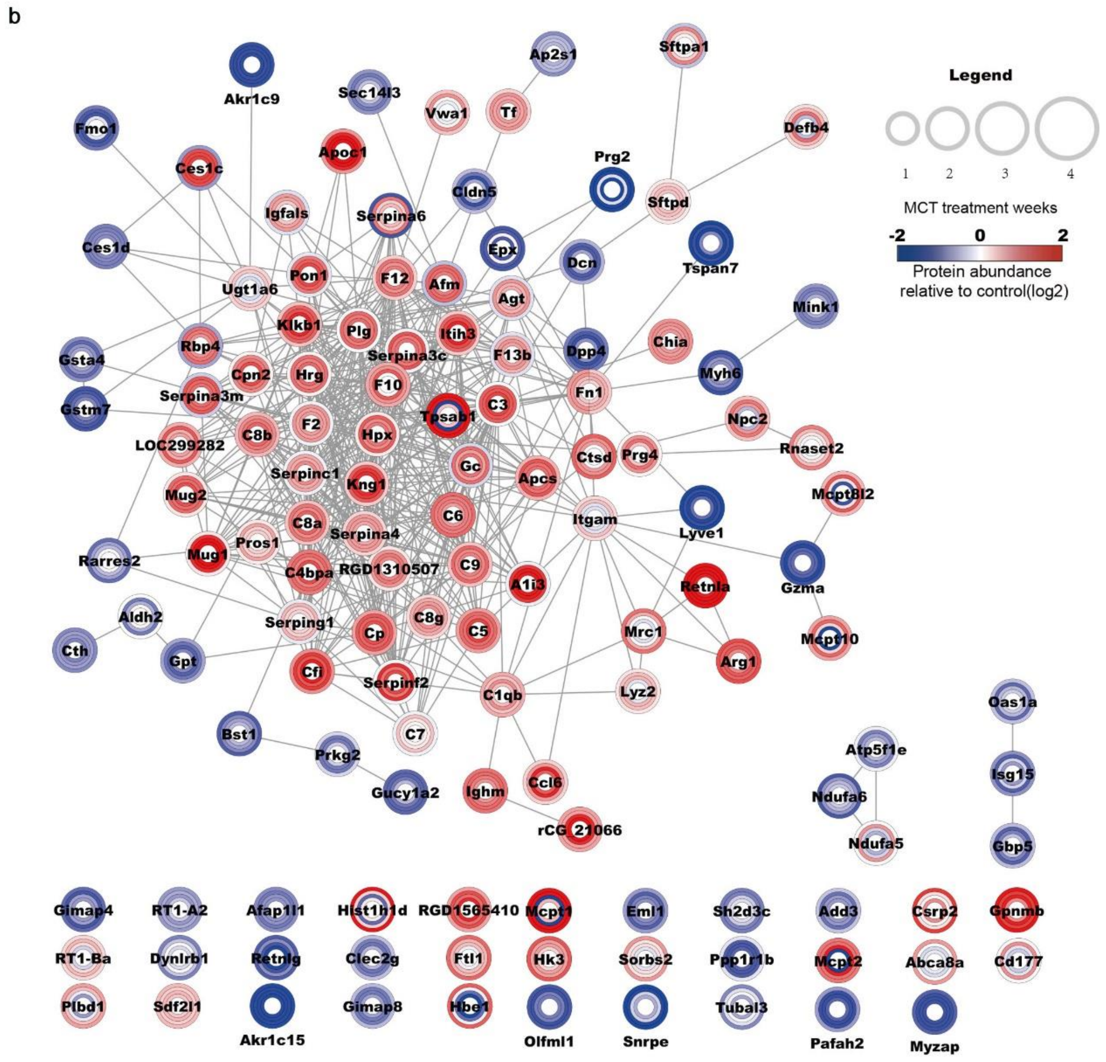

Supplementary Figure S8. Compartment analysis-based protein-protein interaction networks of proteins significantly changed in lung tissues of MCT-treated rats. Proteins with | $\log_2$(FC) | >1.5 and p-value < 0.05 in at least two groups of data or changed continuously for at least three weeks were included. Proteins localized in specific cellular compartment were shown in **a,** while the undermined proteins were shown in **b.**

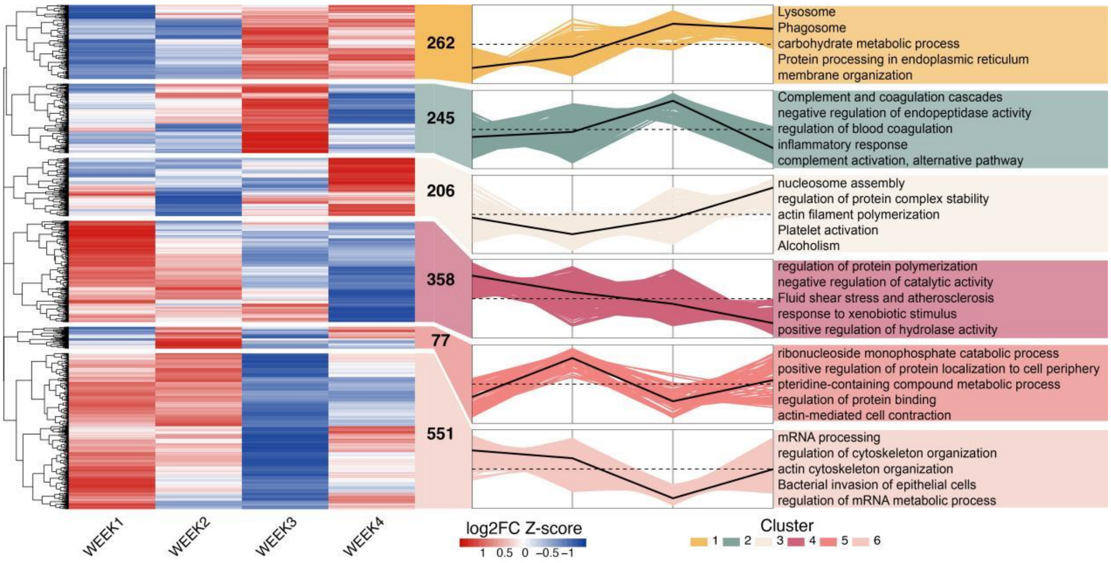

Supplementary Figure S9. Hierarchical clustering of fold changes and pathway analysis of significantly changed proteins in proteomic profiling of lung tissues from MCT model rats. Proteins significantly changed （abs （$\log_2$(fold change)>$\log_2$(1.2), P <0.05) in at least one group are included. Number of proteins within each cluster, fold change profiles and selected pathways are shown.

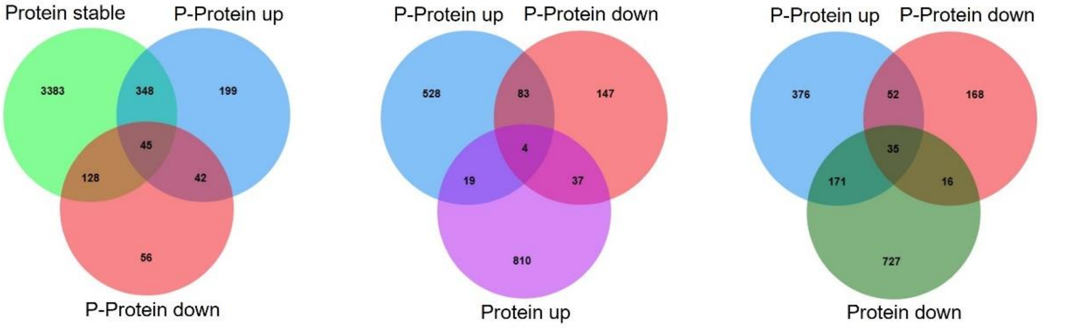

Supplementary Figure S10. Venn diagrams showing the associations between significantly changed phosphoproteins (P-Protein) and proteins quantified in lung tissue from rats treated with MCT for 3 weeks.
