# Supplementary figures and images for: Quantitative Proteomic and Phosphoproteomic Profiling of Lung Tissues from Pulmonary Arterial Hypertension Rat Model
Source: Int J Mol Sci. 2023 Jun 1;24(11):9629. doi: 10.3390/ijms24119629 (PMC10253736; doi:10.3390/ijms24119629)

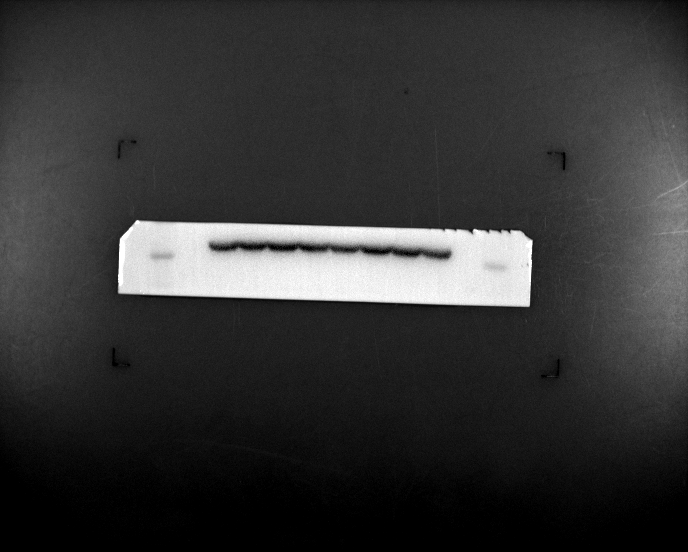

Supplement: Supplementary file 1 [file ijms-24-09629-s001.zip › Original Images for Blots/actin for Week2 Plvap.Tif]

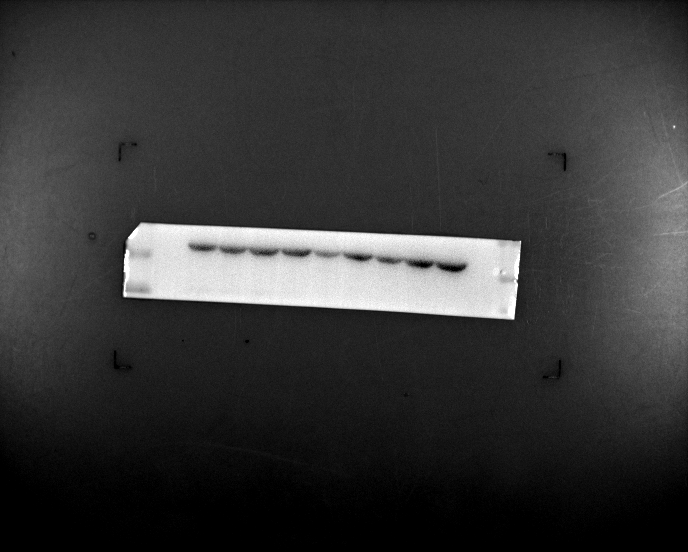

Supplement: Supplementary file 1 [file ijms-24-09629-s001.zip › Original Images for Blots/actin for Week4 Plvap.Tif]

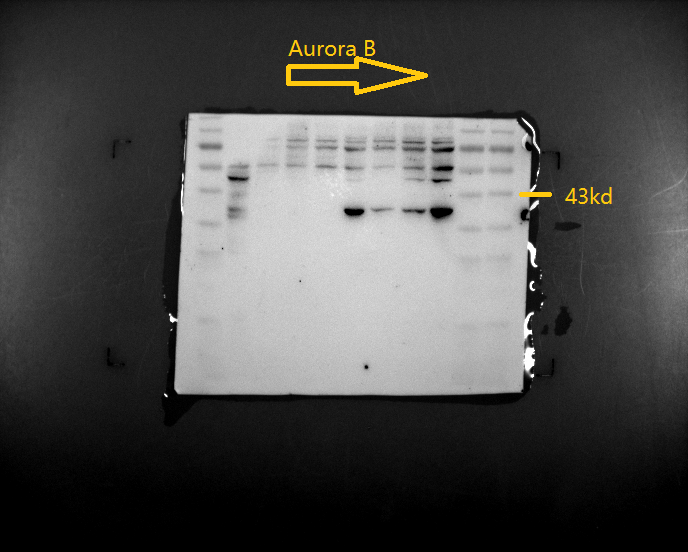

Supplement: Supplementary file 1 [file ijms-24-09629-s001.zip › Original Images for Blots/Aurora B+marker.Tif]

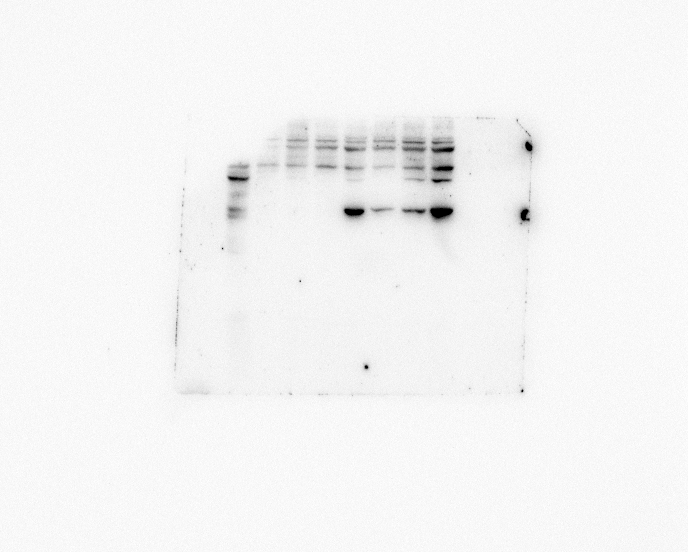

Supplement: Supplementary file 1 [file ijms-24-09629-s001.zip › Original Images for Blots/Aurora B.Tif]

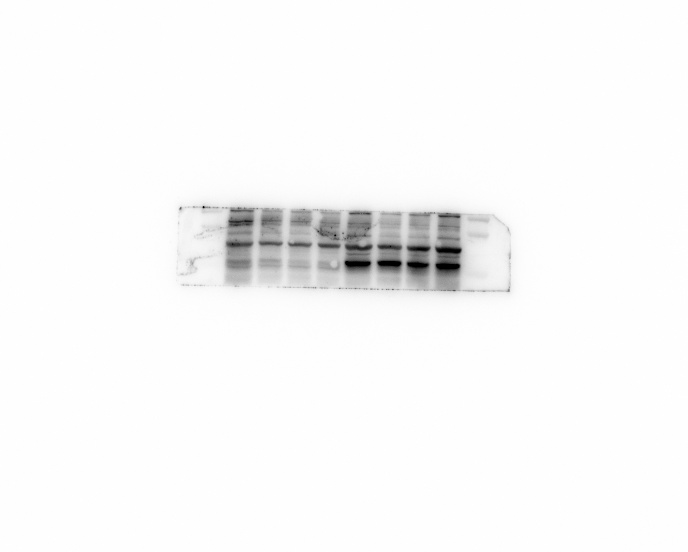

Supplement: Supplementary file 1 [file ijms-24-09629-s001.zip › Original Images for Blots/Ccna2+marker.Tif]

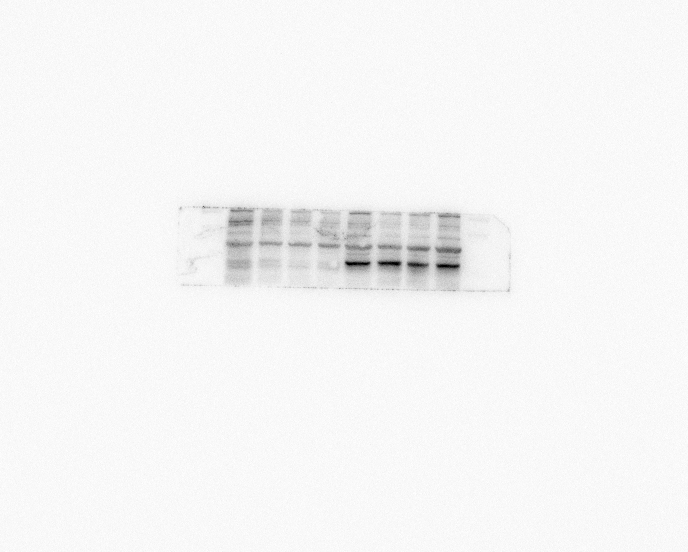

Supplement: Supplementary file 1 [file ijms-24-09629-s001.zip › Original Images for Blots/Ccna2.Tif]

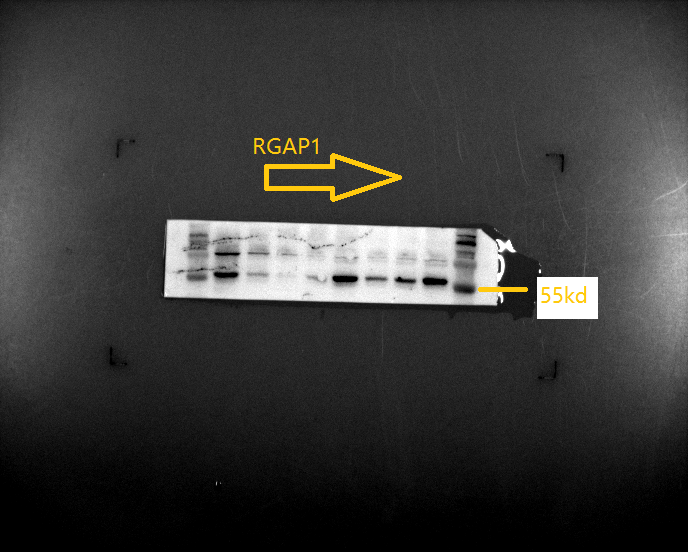

Supplement: Supplementary file 1 [file ijms-24-09629-s001.zip › Original Images for Blots/Racgap1+marker.Tif]

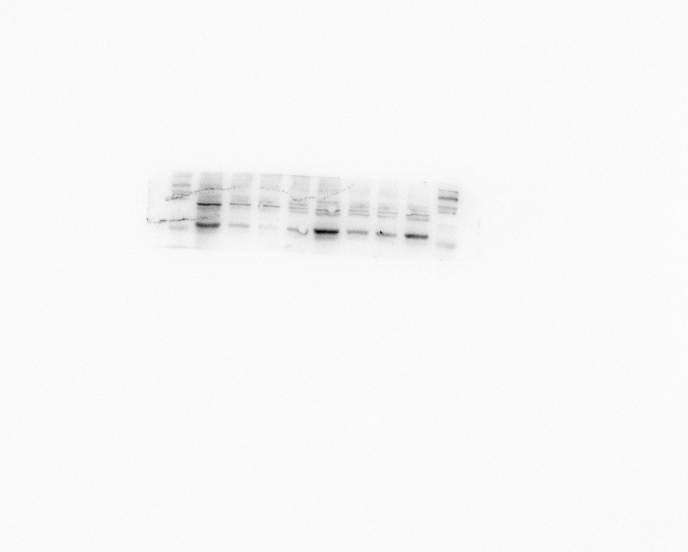

Supplement: Supplementary file 1 [file ijms-24-09629-s001.zip › Original Images for Blots/Racgap1-2.Tif]

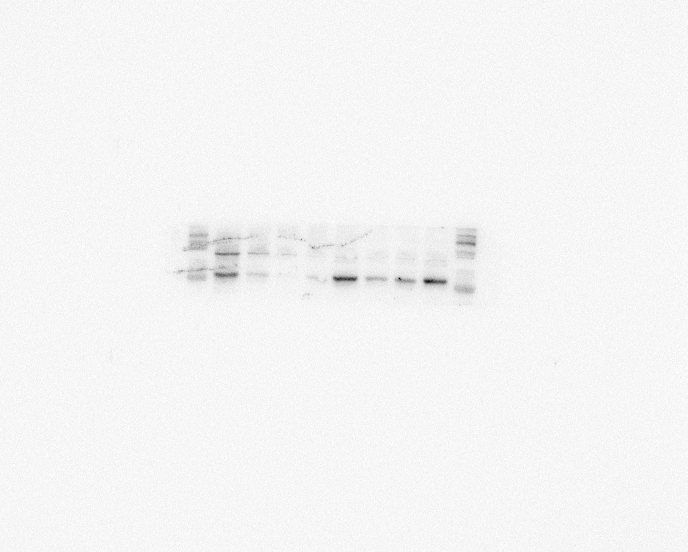

Supplement: Supplementary file 1 [file ijms-24-09629-s001.zip › Original Images for Blots/Racgap1.Tif]

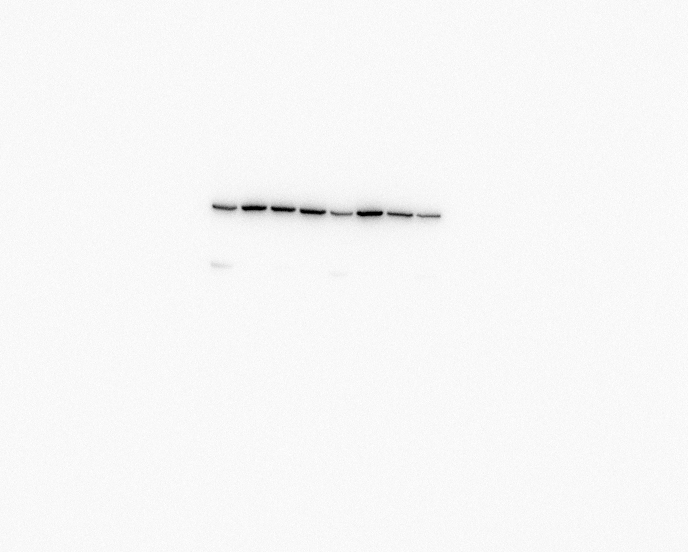

Supplement: Supplementary file 1 [file ijms-24-09629-s001.zip › Original Images for Blots/tubulin for Aurora B.Tif]

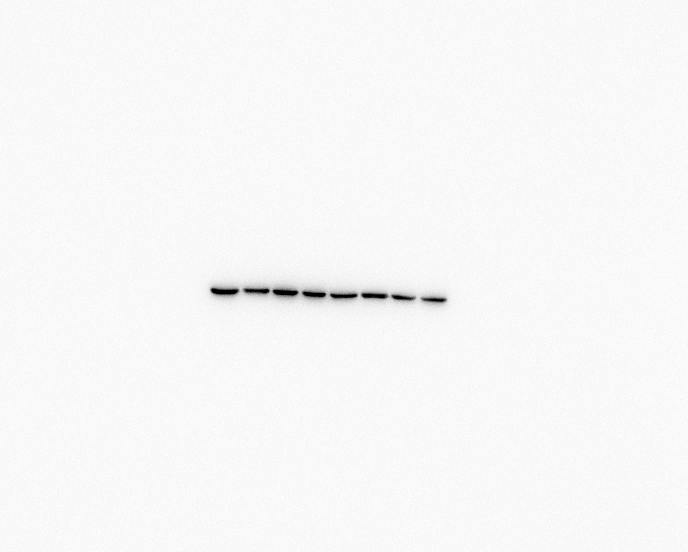

Supplement: Supplementary file 1 [file ijms-24-09629-s001.zip › Original Images for Blots/tubulin for Ccna2 and Racgap1.Tif]

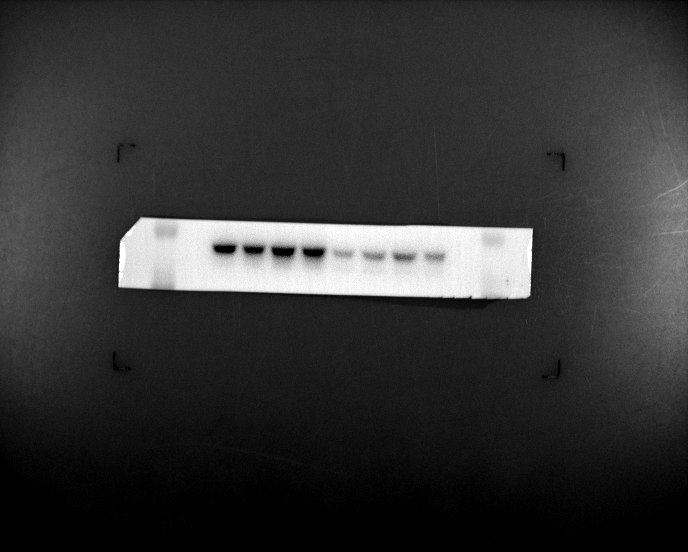

Supplement: Supplementary file 1 [file ijms-24-09629-s001.zip › Original Images for Blots/Week2 Plvap.Tif]

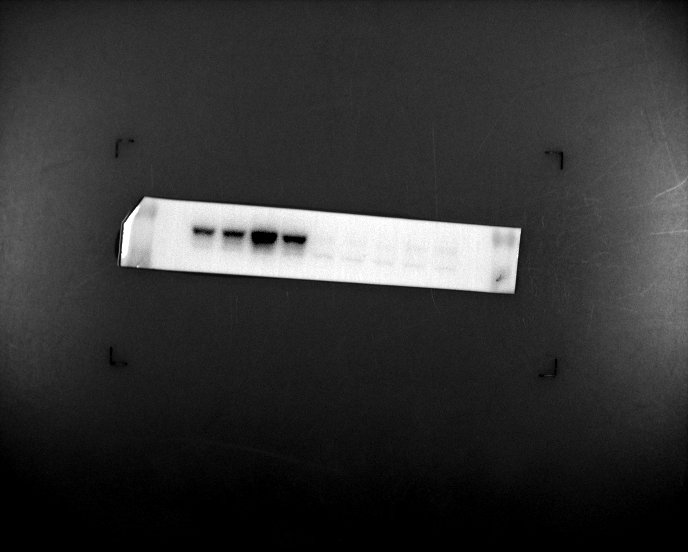

Supplement: Supplementary file 1 [file ijms-24-09629-s001.zip › Original Images for Blots/Week4 Plvap.Tif]
